# Manipulation of cell cycle progression can counteract the apparent loss of correction frequency following oligonucleotide-directed gene repair

**DOI:** 10.1186/1471-2199-8-9

**Published:** 2007-02-06

**Authors:** Julia U Engstrom, Eric B Kmiec

**Affiliations:** 1 Department of Biological Sciences, University of Delaware, Delaware Biotechnology Institute, 15 Innovation Way, Newark, DE 19711, USA

## Abstract

**Background:**

Single-stranded oligonucleotides (ssODN) are used routinely to direct specific base alterations within mammalian genomes that result in the restoration of a functional gene. Despite success with the technique, recent studies have revealed that following repair events, correction frequencies decrease as a function of time, possibly due to a sustained activation of damage response signals in corrected cells that lead to a selective stalling. In this study, we use thymidine to slow down the replication rate to enhance repair frequency and to maintain substantial levels of correction over time.

**Results:**

First, we utilized thymidine to arrest cells in G1 and released the cells into S phase, at which point specific ssODNs direct the highest level of correction. Next, we devised a protocol in which cells are maintained in thymidine *following *the repair reaction, in which the replication is slowed in both corrected and non-corrected cells and the initial correction frequency is retained. We also present evidence that cells enter a senescence state upon prolonged treatment with thymidine but this passage can be avoided by removing thymidine at 48 hours.

**Conclusion:**

Taken together, we believe that thymidine may be used in a therapeutic fashion to enable the maintenance of high levels of treated cells bearing repaired genes.

## Background

A major goal of molecular medicine is to replace or repair a dysfunctional gene in the context of its natural position in the chromosome. A number of avenues of investigation are being taken including those that utilize short oligonucleotides to direct the single base changes. Triplex-helix forming oligonucleotides conjugated to reactive chemical groups and those constructed as bifunctional oligonucleotides continue to show promise in model systems [1-4]. Most of the effort however, centers on the use of modified or unmodified single-stranded DNA oligonucleotides (ssODNs) ranging in length from 25 to 75 bases. The original concept for using ssODNs came from studies in yeast in the 1980s that were pioneered by Sherman and colleagues [see [5]] and adapted to episomal targets in mammalian cells by Campbell *et al. *1989. Gamper *et al. *[6,7] and Liu *et al. *[8] then re-established single-stranded vectors as potential agents for the alteration of chromosomal sequences in eukaryotes.

The mechanism by which oligonucleotides-directed gene repair takes place is still being elucidated. One lab has proposed that transcription-coupled repair alone can account for base exchange or correction [9]. But, the majority of workers believe that DNA replication and DNA repair activities comprise the major pathway of gene repair. This concept was originally proposed by Brachman and Kmiec [10], subsequently extended by Parekh-Olmedo *et al. *[11], and now has been supported by data generated by numerous laboratories [12-17]. This model suggests that actively replicating genomes are more amenable to the hybridization of the ssODNs at the target site because the chromatin is in a more open configuration and the target sequence more accessible. The use of agents such as hydroxyurea [18], ddC [19], or thymidine [14,20,21] that retard the process of DNA replication enable higher levels of gene repair activity by expanding the window of time within which the ssODN can locate its target site.

The role of replication in the gene repair reaction, however, has recently been met with a paradoxical series of events. The initialization of the gene repair reaction results in a plethora of problems for the cell and its metabolism because an ATM-dependent DNA damage response pathway is activated by the transfer of the ssODN [22], confirming earlier observations by Liu [see [23]]. Activated ATM signals the induction of the checkpoint regulator proteins Chk1 and Chk2, which in turn direct cell cycle arrest predominantly in corrected cells. This result provided an explanation for the important observations of Olsen *et al*. [12,13] that the percentage of corrected cells in a population of treated cells decreases as a function of time, presumably because the uncorrected population continues to proliferate while the corrected cells are arrested. If this phenomenon is applicable to primary cells, then the use of gene repair for treating inherited diseases will suffer a severe setback. The goal of this work was to devise an experimental protocol to avert the dilution of corrected cells in a population of treated cells.

As mentioned, the addition of thymidine to cells being targeted for gene repair enhances the frequency with which single base exchange takes place [14,18,21] by synchronizing cells at the G1/S border (referred to as a thymidine block) [24] followed by their release into S phase *en masse*, which provides an increased number of accessible targets for repair. In addition to the increase level of gene repair observed after a thymidine treatment, we show that the agent is able to stabilize the correction frequency over several days, perhaps by inhibiting the replication of the entire cell population and preventing a dilution effect on the corrected cells. We also reveal a novel observation that long-term exposure to thymidine results in the reversion of DLD-1 cells into cellular senescence and that this can be avoided by the removal of the drug prior to this point. Based on this work and earlier observations, thymidine is an attractive reagent for downstream clinical applications due to its ability to elevate and maintain substantial levels of gene repair without inducing severe toxicity *in vitro*.

## Results

A mutant eGFP gene, containing a stop codon in the region encoding the chromophore domain of the protein, was integrated in DLD-1 cells and a clonal isolate consisting of 2–3 copies of the mutated gene was isolated and used as the target for oligonucleotide-directed gene repair [10,18-22,25,26]. A single-strand oligonucleotide (ssODN) consisting of 47 bases with three phosphorothioate linkages on each end, designed to hybridize to the nontranscribed strand of the eGFP gene, was used to reverse the mutation (TAG → TAC),. As previously reported, correction resulting in the restoration of green fluorescence expression can be assessed by flow cytometry analyses at various times following ssODN introduction [18].

A variety of reagents have been tested with the aim of elevating the frequency of gene repair in mammalian cells. Among these are drugs that induce double-stranded DNA breaks (ds breaks) and chemicals that form reversible adducts within the helix [27]. In most cases, DNA damage results in a slowing of the cell cycle and an expanded S phase and so, reagents that simply reduce the rate of DNA replication without causing double strand breaks have also been found to improve the efficiency of gene repair [10,19]. These agents are able to increase correction efficiency by expanding the window of time within which the ssODN gains access to the target site [10,19]. Thus, our testable prediction is that a reagent such as thymidine, which increases the cellular levels of dTTP via a depletion of dCTP, resulting in a reduction in the rate of DNA replication [24], will increase gene repair activity.

### Pre-incubation with thymidine elevates gene repair by increasing the number of cellular targets amenable for correction

**Figure 1:**
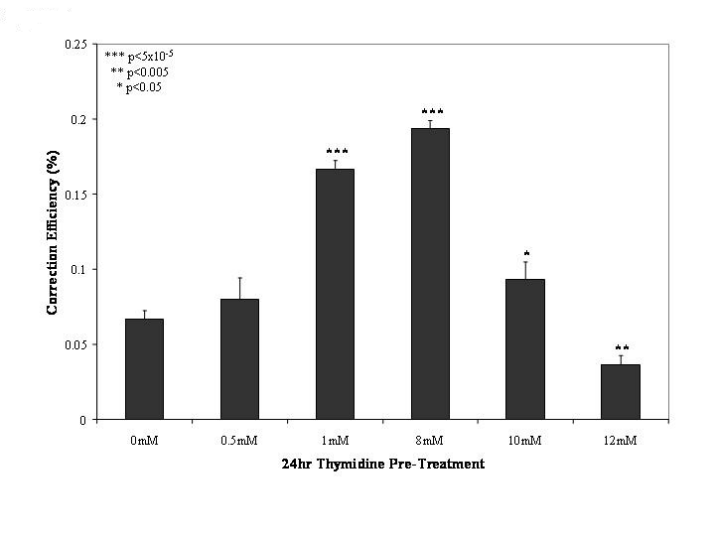
***A 24 hour pre-treatment with thymidine enhances gene repair levels***. Various concentrations of thymidine were added to a growing culture of DLD-1 cells 24 hours prior to the introduction of 1 μM oligonucleotide (EGFP3S/47NT). Cells were allowed to recover for 48 hours after electroporation and were harvested for FACS analysis. Correction efficiency is reported as the percent of live green fluorescent cells in the total cell population (50,000 per experiment); mean and standard deviations were calculated based on values obtained from 3–4 samples per treatment. T-tests were performed comparing the 1 mM and 8 mM thymidine pre-treatment to the 0 mM sample (***p < 5 × 10^-5^).

Thymidine was added at various concentrations to the DLD-1 mutant eGFP cell cultures 24 hours prior to the introduction of the ssODN (EGFP3S/47NT) and correction levels were determined 48 hours after electroporation by flow cytometry (Figure 1). The results show a maximal response at concentrations of 1 and 8 mM thymidine, with a significant increase of about 3 fold from the non-treated sample. The decrease in correction efficiency as seen at higher concentrations of thymidine (10 and 12 mM) is most likely due to the enhanced thymidine cytotoxicity reported previously in DLD-1 cells due to their mutation in Msh6 [28]. Although these results differ from the observation of Wu *et al*. [14], who stated that the incubation of thymidine prior to the introduction of the oligonucleotide actually lowered gene repair in HeLa cells containing >50 copies of the target gene, our results are consistent with other reports that a thymidine pre-treatment is able to elevate gene repair several fold over basal levels.

**Figure 2:**
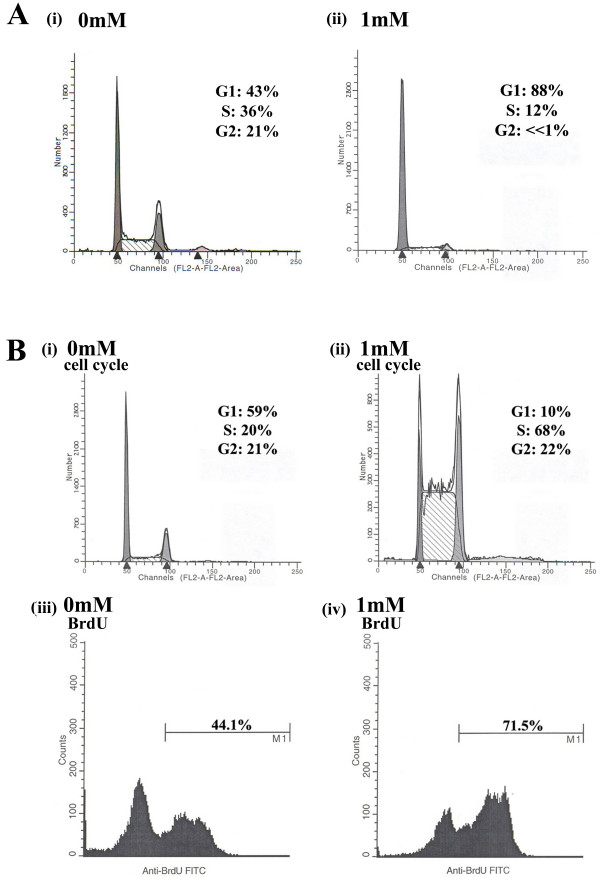
***Pre-treatment with thymidine arrests cells in G1 and once removed, allows a synchronous entry into S phase***. **(A) **Thymidine was added at 1 mM (ii) or 0 mM (i) for 24 hours, cells were then harvested and processed for cell cycle analysis, representing the cell cycle profile at the time of oligonucleotide electroporation. **(B) **Cells were treated for 24 hours as in A, but were washed 2× with PBS at 24 hours and released into fresh media for 8 hours, at which time they were either collected for cell cycle analysis (i and ii) or were stained for BrdU incorporation (iii and iv) to measure their replicative state. The position of M1 (BrdU+ marker) was determined by a BrdU(-) control, not shown, and percents indicate the number of cells that are positive for BrdU in the total sample size (25,000 cells).

It is widely known that the addition of thymidine to a mammalian cell culture leads to a synchronization of cells in the G1 phase of the cell cycle or at the G1/S border [29,30]. When cells are released from the thymidine block, a large fraction of the population proceeds into S phase, resulting in an increase of ideal cellular targets for ssODN site association and thus, gene repair activity appears maximized [19,26]. As shown in Figure 2A, the addition of 1 mM thymidine restructures the cell cycle profile such that the majority of cells are primarily stalled in G1, minimally in S phase, and with only a small fraction in G2 (Figure 2A: ii). By 8 hours after the release of the thymidine block, the cells begin to transition into S phase (Figure 2B: ii), while the non-treated samples remain predominantly at the same cell cycle proportions from the time of oligo addition to 8 hours following electroporation (Figure 2A: i and Figure 2B: i). At 8 hours, a majority of the cells released from the thymidine treatment contain actively replicating chromosomes (Figure 2B: iii), as assayed by BrdU incorporation. Under these conditions, the number of actively replicating cells is almost twice as high as the level seen in cells that have not been pretreated with thymidine (Figure 2B: iv vs. 2Biii), an observation that could reflect the increased number of cells in S phase. Thus, the addition of thymidine and its subsequent removal stimulates gene repair activity by enabling more cells to be in S phase when the ssODN initializes the gene repair reaction.

### Thymidine is able to stimulate and maintain substantial levels of gene repair

**Figure 3:**
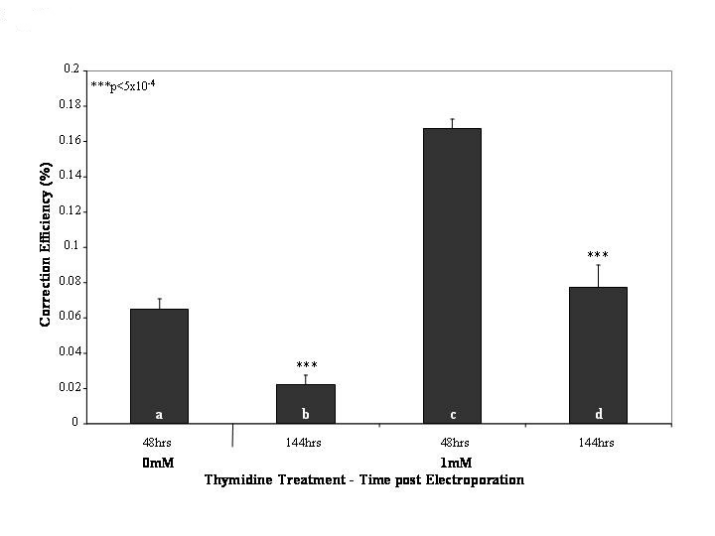
***Over time, gene repair levels diminish regardless of thymidine pre-treatment***. Cells were treated either with no thymidine (*a *and *b*) or with 1 mM thymidine (*c *and *d*) for 24 hours prior to electroporation of the oligonucleotide. The cells were then allowed to recover for 48 hours (*a *and *c*) or 144 hours (*b *and *d*), at which points they were harvested and measured by FACS for eGFP expression. The correction efficiency reports the percent of the total number of cells (50,000 in each sample) that are live and positive for eGFP fluorescence. The mean and standard deviations were calculated based on the results from 4 individual samples of each treatment/time point; T-tests were performed comparing samples *a *to *b *and *c *to *d *(***p < 5 × 10^-4^).

After observing the increase in gene repair levels at 48 hours following a 24 hour thymidine pre-treatment, we wanted to examine if this increase remains stable over time, beyond the 48 hour incubation. For this study, we repeated the experiment outlined in Figure 1 using a single concentration of thymidine (1 mM) during the pre-incubation phase and measured correction levels at 48 and 144 hours after ssODN electroporation. Although the initial stimulation is seen at 48 hours post electroporation, Figure 3 illustrates an important phenomenon in which the absolute level of correction decreases with time, corroborating the initial finding reported by Olsen *et al. *[12,16]. As shown, a statistically significant decrease in repair levels is seen between the 48 and 144 hour time points (approximately 3-fold) for both the pretreated (Figure 3: bar *d *vs. *c*) and untreated (Figure 3: bar *b *vs. *a*) samples. It has been suggested previously by our group that this lack of maintenance of the correction efficiency is due to the induction of the DNA damage response pathway and its subsequent activation of the checkpoint proteins Chk1 and Chk2. Hence, a "dilution" of the corrected cells is seen as the non-corrected cell population proliferates at normal rates while the corrected cells appear stalled [22].

**Figure 4:**
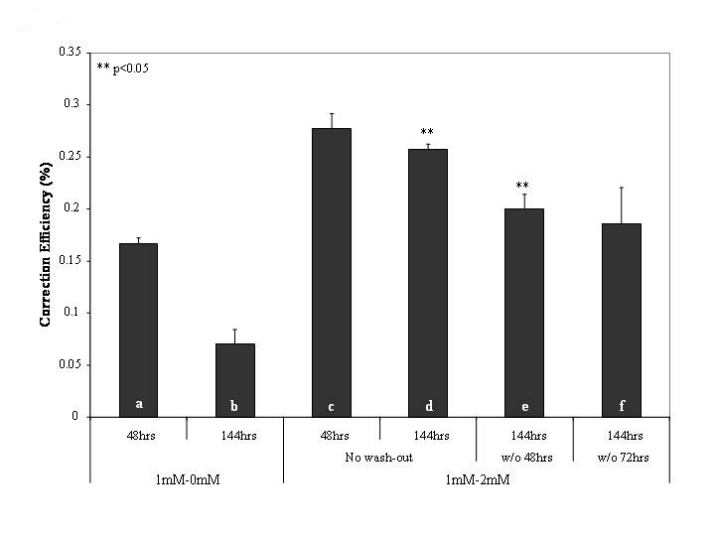
***Combining the 1 mM thymidine pre-treatment with a 2 mM post-treatment retains high levels of gene repair***. Cells were treated with 1 mM thymidine for 24 hours, electroporated with 1 μM EGFP3S/47NT oligo, and recovered in complete medium without thymidine (*a *and *b*) or in the presence of 2 mM thymidine (*c *through *f*) for 48 hours (*a *and *c*) or for 144 hours (*b *and *d-f*). At 48 and 72 hours, sample *e *and *f*, respectively, were washed 2× with PBS and fresh medium was added that did not contain any thymidine (termed "w/o" for "washed out"); these cells were collected at 144 hours and analyzed by FACS for eGFP fluorescence. Correction efficiency depicts the percentage of live green fluorescent cells in the total number of cells counted (50,000 per sample); means and standard deviations were calculated based on the results of three to four independent experiments. T-tests were performed comparing *b *to *d *and *e *(**p < 0.05).

Based on this observation, we wanted to examine the idea of controlling replication of the entire cell population as a means of preventing the decrease of correction levels over time. Our reasoning behind this was to transiently inhibit the replication and division of non-corrected cells until the corrected cells were able to resume normal replication. Accordingly, we wanted to utilize thymidine and its ability to stall cell replication as a tool to slow down the entire cell culture, in hopes of maintaining the high initial levels of correction for extended periods of time. To address this aim, we treated the cells with 1 mM thymidine for 24 hours prior to the introduction of the 47-mer EGFP3S/47NT and then divided the samples into three distinct groups. The first group of cells was grown in the absence of thymidine for either 48 or 144 hours (Figure 4: *a *and *b*) while the second group was grown for 48 or 144 hours in the presence of 2 mM thymidine (Figure 4: *c *and *d*). The last group was grown in thymidine for only 48 or 72 hours after electroporation (Figure 4: *e *and *f*, respectively), at which point the thymidine was washed out (indicated as "w/o") and the incubation continued for a total of 144 hours in complete medium. The combination of a 1 mM pre- and a 2 mM post-treatment was optimized to produce first an initial increase in repair frequency followed by a sustained level of this with minimal cytotoxicity, data not shown. When no thymidine was present in the post-electroporation cultures, the correction efficiency decreased significantly between the 48 and 144 hour time points, coinciding with the data in Figure 3. In contrast, when 2 mM thymidine was added to the cultures following electroporation, the correction efficiency was largely maintained between 48 and 144 hours at levels significantly above those for the non-treated samples at corresponding time points. In a similar fashion, by removing thymidine after only a 48 or 72 hour incubation, correction efficiencies still remained significantly higher at 144 hours than with out any thymidine post-treatment. Taken together, these data suggest that the presence of thymidine in the post-electroporation reaction results in a maintained level of gene repair for an extended period of time.

### Thymidine addition induces cell senescence in DLD-1 cells under certain reaction conditions

**Figure 5:**
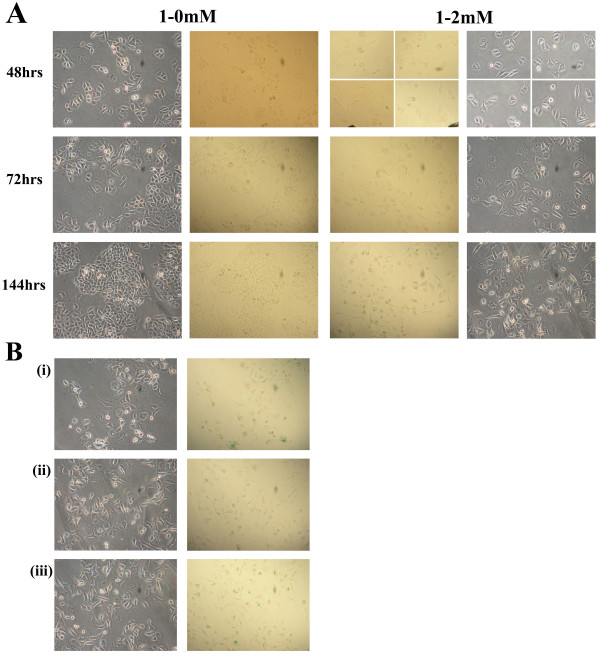
***Prolonged exposure to thymidine results in cellular senescence***. **(A) **Following a 1 mM thymidine 24 hour pre-treatment, cells were electroporated and released into complete medium (1-0 mM) or medium supplemented with 2 mM thymidine (1–2 mM). At 48, 72 and 144 hours, cells were stained for the senescent marker β-galactosidase. The four quadrants for the 1–2 mM sample at 48 hours are combined images obtained from four different fields of view, due to the low confluency of the sample. **(B) **Images depict varying views and different wells of the 1–2 mM thymidine treated cells at 144 hours. All images are at 20× magnification and counts of individual cells in a representative field were performed to evaluate the degree of senescence induction.

**Figure 6:**
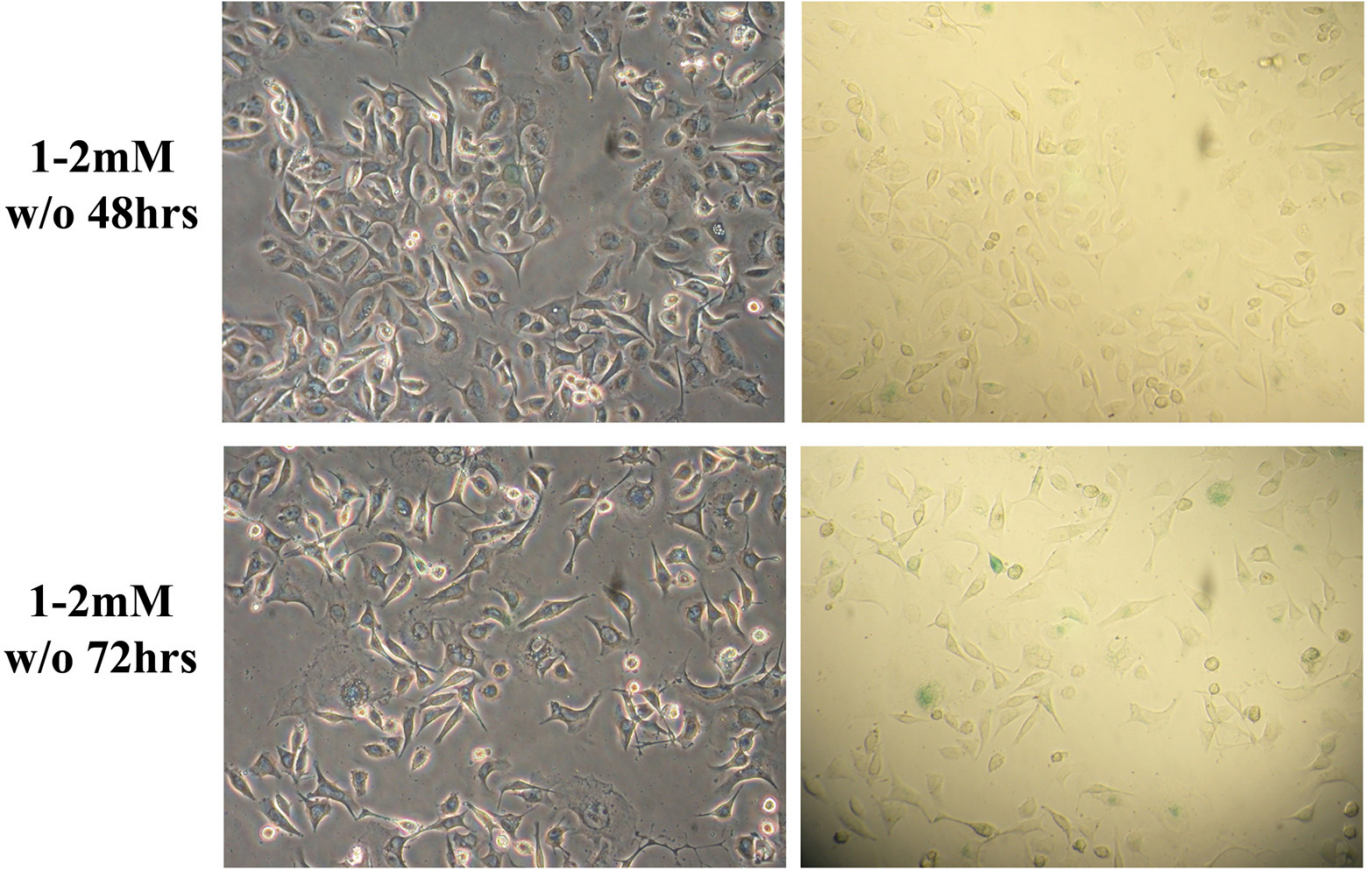
***Removal of the 2 mM thymidine post-treatment at 48 hours, but not at 72 hours, prevents cellular senescence***. Cells were treated with 1 mM thymidine prior to electroporation and then recovered in 1 mM thymidine. At 48 hours and 72 hours, as indicated, cultures were washed 2× with PBS and fresh medium was added ("w/o": washed out). At 144 hours, cells were assayed for senescence-associated β-galactosidase activity. All images are at 20× magnification.

After extended culturing in the presence of thymidine, we noticed morphological changes within our cultures that resembled a senescent phenotype, including flattened cell morphology with elongated cellular processes and enlarged nuclei [31]. To confirm these cellular changes, cultures were stained for senescence associated β-galactosidase (SA β-gal) expression [32,33]. In Figure 5A, representative fields of cells treated with thymidine under two different conditions are shown at 48, 72 and 144 hours after introduction of the ssODN. No evidence of senescence is observed in the 48 or 72 hour cultures under either condition (1-0 mM or 1–2 mM). However, cells pretreated with 1 mM thymidine and incubated in 2 mM thymidine after electroporation (1–2 mM) develop a senescent phenotype within 144 hours, as evident by the presence of blue stain in approximately 50% of these cells. In Figure 5B (i, ii, and iii), multiple images obtained at the 144 hours under the 1–2 mM thymidine condition are presented, further highlighting the positive SA β-gal expression. The left-hand panels, showing cell morphology, were photographed under phase contrast while the panels on the right, aimed to enhance the blue color, were photographed with bright field illumination. Here, the evidence for certain reaction conditions inducing cellular senescence is more compelling and this transition to a senescent phenotype could further explain the maintenance of gene correction over 144 hours with the 1–2 mM thymidine treatment combination. In contrast, Figure 6 presents cells incubated for 144 hours with thymidine having been washed out at 48 or 72 hours, post-electroporation. Here, we observe a minimal number of senescent cells at the 48 hour wash-out (less than 10% of the cells) but a significant number is observed when the thymidine is washed out at 72 hours (about 38% of the culture), suggesting that the transition to wide-spread senescence most likely occurs shortly after this point. Furthermore, it is important to note that corrected cells are neither apoptotic nor senescent (Ferrara et al., submitted), suggesting that the transition to the apoptotic state is simply as a result of the thymidine incubation. In addition, prolonged treatments with thymidine did not show evidence of cell death, as determined by a MTT (3-(4,5-Dimethylthiazol-2-yl)-2,5-diphenyltetrazolium bromide) cell viability assay in which the level of production of the purple formazan remained unchanged during the incubation (data not shown).

### Prolonged incubation with thymidine leads to a slow-down in DNA replication and cell division

**Figure 7:**
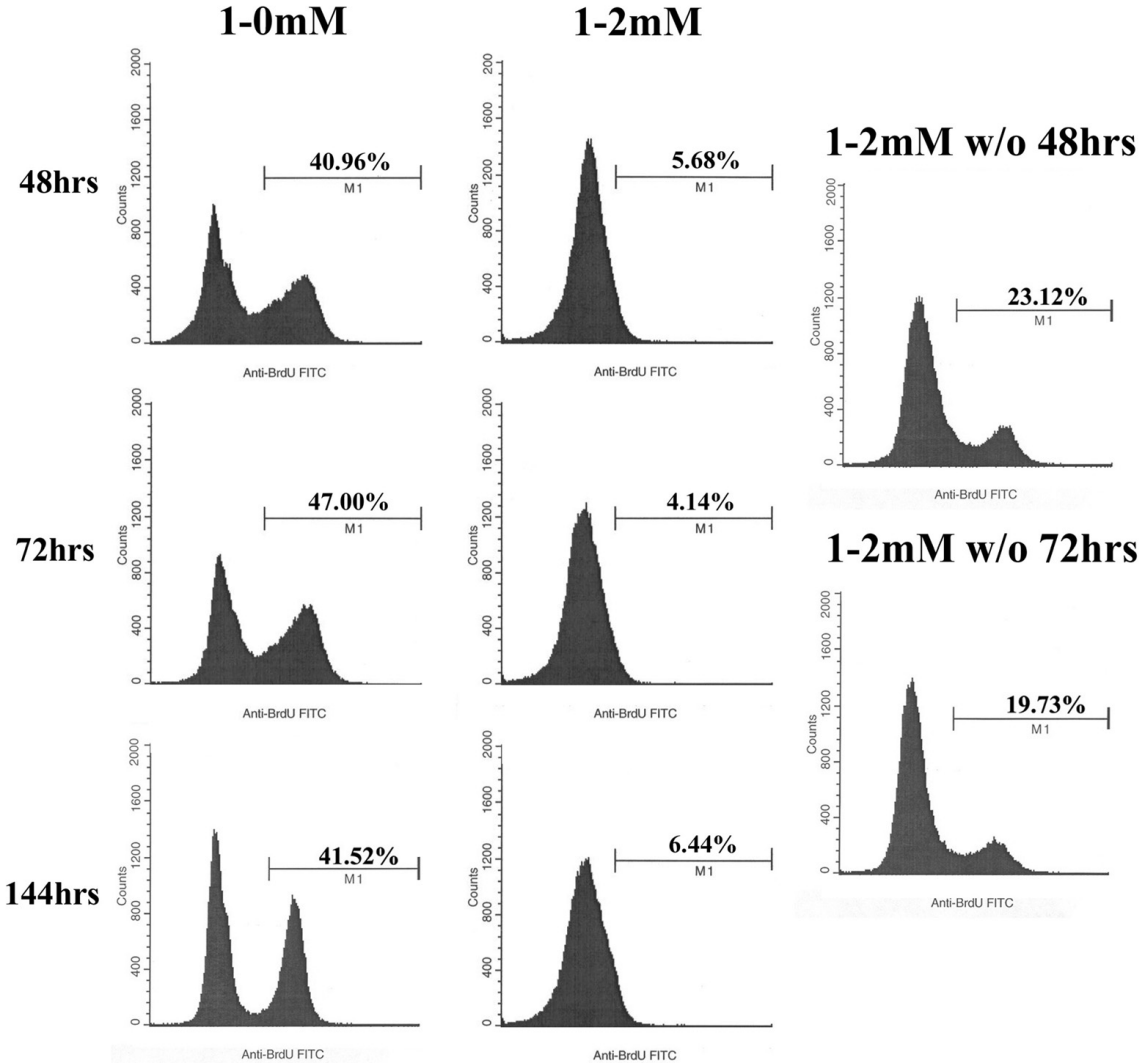
***Cells are able to resume replication after the removal of 2 mM thymidine during recovery***. Cells were treated with 1 mM thymidine for 24 hours, electroporated, and recovered in 2 mM thymidine (1–2 mM) or without thymidine (1-0 mM). At 48, 72, and 144 hours, the culture media was supplemented with BrdU and cells were later assayed by FACS for its incorporation. The 1-0 mM culture reached confluency by 96 hours and was passaged once prior to the 144 hour time point. The w/o ("wash out") samples received 2 mM thymidine in the recovery phase, which was subsequently washed out at 48 and 72 hours, respectively, and later assayed at 144 hours for BrdU incorporation. Position of M1 (BrdU+ marker) was determined by a BrdU(-) control, not shown, and percents indicate the number of cells that are positive for BrdU incorporation out of the total population (25,000 cells).

As shown in Figure 2A, the exogenous addition of thymidine to a DLD-1 cell culture results in an accumulation of cells in G1 or at the G1/S border. In effect, thymidine reduces the overall rate of DNA replication in a population of cells by synchronizing them into G1 and preventing their entry into S phase. Thus, the presence of thymidine in a gene repair reaction could simply be maintaining the relative percentages of corrected and non-corrected cells between 48 and 144 hours by inhibiting the replication of the entire cell population and there by, preventing the dilution of the corrected cells. To examine this possibility, we measured the capacity of cells to incorporate BrdU, which would indicate the percent of cells undergoing active replication at 48, 72, and 144 hours. When the population was pretreated with thymidine for 24 hours followed by a removal of the thymidine at the time of electroporation (Figure 7: 1-0 mM), replication activity remained fairly constant overtime. In contrast, when the pretreatment was followed by sustained incubation in thymidine following electroporation (Figure 7: 1–2 mM), replication activity dropped dramatically and little incorporation of BrdU was evident. Thus, it seems likely that the maintained correction efficiency seen between 48 and 144 hours under the latter condition (1–2 mM) is due to an overall inhibition of DNA replication and in part by the entry into cellular senescence, as seen in Figure 6. However, when thymidine was washed out at 48 or 72 hours (Figure 7: 1–2 mM w/o 48 hrs and 1–2 mM w/o 72 hrs, respectively), and cells allowed to resume growth in complete medium, DNA replication resumed, reaching approximately half the normal level of activity by 144 hours of incubation. More importantly, this washout protocol preserved high levels of correction efficiency between 48 and 144 hours (see Figure 4). Therefore, the presence of thymidine during the early times of the gene repair reaction appears to help maintain correction levels without irreversibly disabling the capacity of the cell to resume DNA replication.

**Figure 8:**
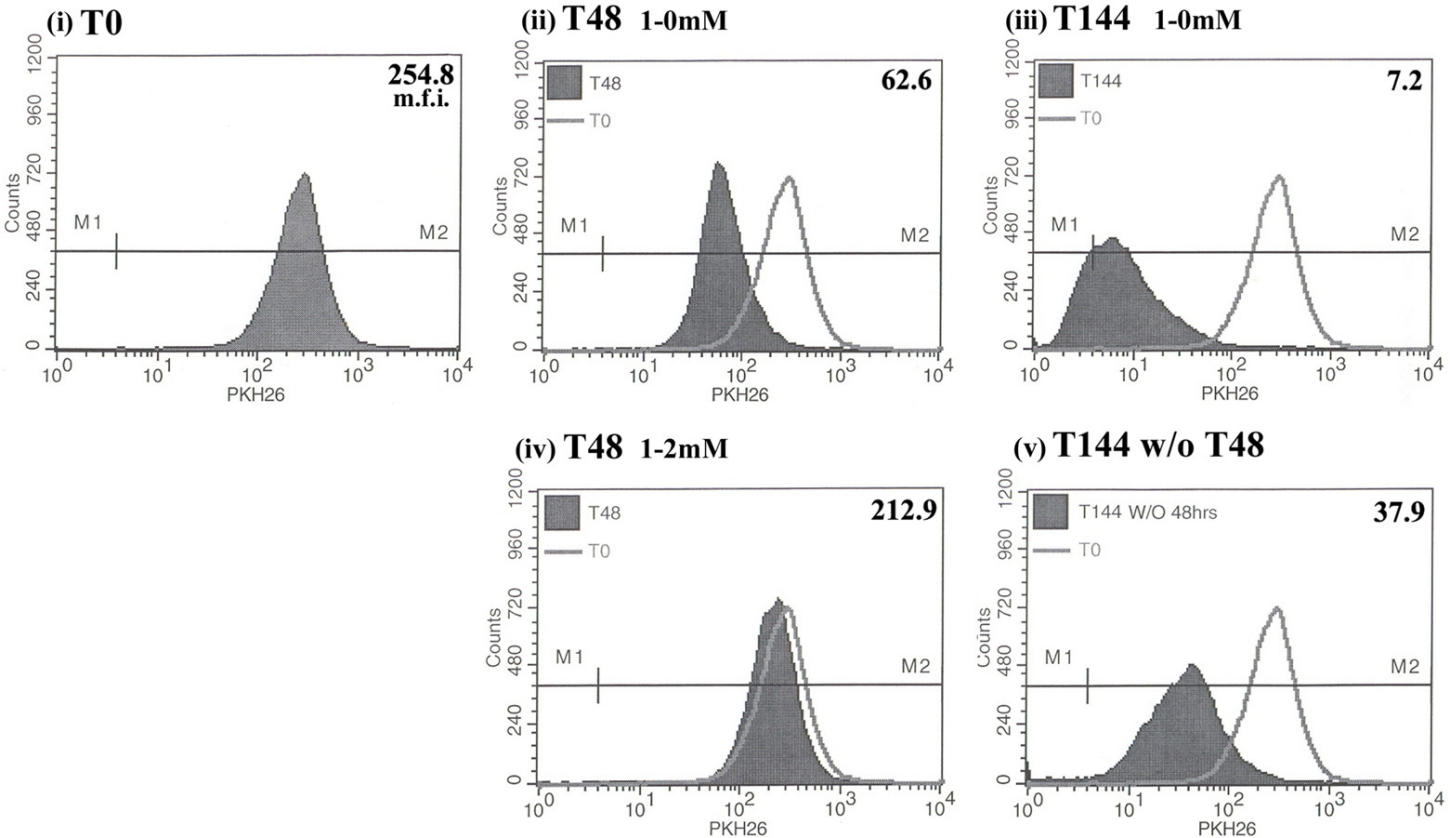
***Following the removal of 2 mM thymidine from the post-treatment, cells are able to resume cell division at rates comparable to no treatment samples***. Following a 24 hour 1 mM pre-treatment with thymidine, cells were collected and stained with the cell-membrane dye, PKH26, and then released into medium supplemented with 2 mM thymidine (iv and v) or complete medium (ii and iii). Fluorescence of the PKH26 dye was measured in a subset of the cells immediately following staining and prior to re-plating to obtain an initial fluorescence value (i: T0); subsequent samples were removed at 48 (ii and iv) and 144 hours (iii and v) following re-plating for the same analysis. Sample (v) was washed 2× with PBS at 48 hours, received fresh medium and analyzed at 144 hours. Values indicate the mean fluorescence intensity (m.f.i.) of the population at each time point (shaded peak); non-shaded graph represents the fluorescence levels at T0 and filled in graphs represent the fluorescence intensity at the indicated times. M1 is the boundary for the PKH26(-) samples and M2 indicates PKH26(+) fluorescence.

In order to confirm these results and to evaluate the potential residual effects if thymidine on the cell cycle following the wash-out protocol, we utilized an assay to determine division rates of cells. PKH26, a fluorescent cell label that binds irreversibly within the lipid region of cell membranes, was used to determine rate and extent of cell proliferation following the removal of the thymidine post electroporation. As cells divide following intercalation of the dye, each resulting daughter cell receives half of the fluorescent dye during subsequent rounds of cell division [34,35]. Thus, by using flow cytometric analysis to measure the change in fluorescence intensity of PKH26, we are able to estimate the number of divisions a cell population has undergone within a given period. At time zero (T0), or at the time of electroporation following a 1 mM thymidine pre-treatment, cells were labeled with PKH26 and the starting mean fluorescence intensity (m.f.i.) was found to be 254.8 (Figure 8: i). At 48 hours following staining, cells that received no thymidine in the post treatment (Figure 8, ii) displayed a shift in their mean fluorescence intensity to 62.6, corresponding to two cellular divisions, and again at the 144 hour point (Figure 8: iii), indicating an additional three cell divisions (total of 5 within the 144 hours). In contrast, with thymidine in the post treatment for 48 hours (Figure 8: iv), there was an insignificant change in the mean fluorescent intensity, indicating that no cell division had occurred. Following the removal of thymidine at 48 hours, the cells underwent 2.5 cell divisions by 144 hours (Figure 8: v), as determined by the change in the mean fluorescence intensity from 48 hours. Thus, it seems as if washing out thymidine at 48 hours can enable cell division to resume at a rate nearly that of the 1-0 mM counterpart. Combined with the BrdU data, a case can be made that the maintenance of correction efficiency is accompanied by DNA replication and normal cell division processes following the removal of thymidine.

## Discussion

Drury and Kmiec [36] demonstrated the importance of target association in the gene repair reaction and suggested that the rate-limiting step is the pairing of the ssODN to the genomic site. Thus, we assume that strategies aimed at increasing correction levels might be facilitated by events that enable more efficient oligonucleotide binding. Several agents have been employed to achieve this goal including trichostatin A (TSA), which is a potent histone deacetylase inhibitor [37]. When added to cell cultures following ssODN delivery, TSA was able to increase gene repair up to 10-fold, most likely by altering the chromosome structure and enabling oligonucleotide binding [38]. Slowing down the process of DNA replication by extending S phase or synchronizing cells in S is another way to enhance the frequency of gene repair [14,18,19,39].

Our current work focused on examining this second manipulation in greater detail by studying more carefully the function of S phase in gene repair. During this phase of the cell cycle, chromatin is in a more open conformation, accessible to proteins/DNA complexes that undergo a search for homology and interact at specific sites within the genome. In this study, we confirm that incubating cells with thymidine leads to elevated gene repair levels by synchronizing the cells along the G1/S border and enhancing the total number of amenable targets when, after release, the cells cycle into S phase in the presence of the oligonucleotide [10,22]. We also show that the inherent loss of gene correction with time [12] can be counterbalanced by inhibiting the replication of the entire cell population, and preventing the "dilution" of the repaired population of cells by the unimpeded non-corrected cell population. Lastly, we present a novel finding that shows that the long term effect of thymidine treatment can result in the induction of cellular senescence.

Treatment with thymidine leads to an increased level of cellular dTTP, ultimately resulting in a feedback inhibition of ribonucleotide reductase. Subsequently, cells are starved of dCTP, due to the allosteric inhibition of the reduction of CDP [40], resulting in a slowing of DNA replication and an accumulation of cells that traverse S phase (referred to as a thymidine block [24]. By incubating DLD-1 cells in 1 mM thymidine for 24 hours, we were able to stall approximately 90% of the cell population at the G1/S border. Following the introduction of the oligonucleotide, the block was removed and the cells were allowed to enter S and G2 in the presence of the ssODN. The high repair efficiency seen with thymidine pre-treatment was most likely due to the increased number of accessible target sites that in S (3 times more cells than in the non-synchronized cells) or cycling through S within the first 8 hours following electroporation. Upon removal of thymidine, cells were able to once again replicate at high levels further indicating the increased number of cells in S phase. Thus, our data indicate that elevated levels of correction are mostly due to an increase of cellular targets in S phase, whose open chromatin structures facilitate ssODN association and ultimately gene repair events.

These results on the importance of S phase in the ssODN-coupled repair differs from the conclusions drawn in Wu *et al. *[14] that increasing the proportion of cells in S phase does not enhance repair levels. Our data contrast these observations by identifying S phase as the critical cell cycle stage in which gene repair occurs. We find that the highest overall gene repair was evident at 8 hours following the ssODN introduction, for both 1 mM thymidine pre-treated and non-treated cells (0.66% and 0.12%, respectively; see Additional file 1). In addition, the maximal value occurred following the 1 mM thymidine pre-treatment, coinciding with the highest percent of cells in S phase at 8 hours. When cells were pre-treated with thymidine and then sustained in thymidine for 8 hrs following ssODN electroporation, gene repair decreased to only 0.21% by this time point, concurrent with an altered cell cycle profile in which a lower percent of cells were in S phase and a higher percent remained at G1 or in early S (see Additional file 2). However, when thymidine was simply added after ssODN introduction (without a pre-treatment), gene repair levels and cell cycle profiles remained remarkably unchanged, as compared to the non-treated non-synchronized cells. Thus, our results support the aforementioned observation that highlights the importance of S phase in the gene repair reaction. What is interesting to note, however, is the fact that although the gene repair is lower when thymidine is used in both the pre- and post-treatment, its repair level remains constant from 8 to 48 hours while a decrease is observed when thymidine is removed following the addition of the ssODN. Ultimately, this change in repair frequency results in the *appearance *of higher levels of gene repair following a 1–2 mM thymidine treatment than a 1-0 mM at 48 hours following ssODN introduction (see Figure 4, bar *a *and *c*).

Recently, it was reported that the repair frequency decreases as a function of recovery time following ssODN introduction, and this lack of maintenance of the repair frequency is due to the selective recovery and proliferation of non-corrected cells [12,22]. Although it is unclear whether the corrected cells are arrested in late S phase [22] or at the G2/M border [12], it is apparent that the single-stranded DNA oligonucleotide activates a damage response pathway through the upregulation and autophosphorylation of ATM [11,22,41]. This activation, in turn, leads to a phosphorylation of the downstream checkpoint kinases, Chk1 and Chk2 at Ser317 and/or Ser 345 and at Thr68, respectively [22,42-45]. Due to the induction of the damage response and the specificity of the Chk1 and Chk2 activation within the corrected cells, these cells are preferentially stalled in the cell cycle and do not divide. In contrast, the non-corrected cells do not uniformly contain activated Chk1/Chk2 and thus, no halt of cellular replication is observed [22]. Because of this lingering cell cycle block, the overall correction frequency appears to decrease as the non-corrected cells resume normal replication while the vast majority of corrected cells become arrested. In contrast to our system in which correction efficiency is measured among a background of non-corrected cells, protocols that utilize selection to isolate corrected cells, in effect, exclude all other cells from the population, only allowing corrected non-arrested cells to continue to grow. Because of this, non-corrected cells are not able to out compete and over grow the corrected cells, and thus, a decrease in gene repair is not evident [46]. In a more biologically relevant target, in which there is no such selective pressure, however, it is imperative to accommodate and respond to the varying replicative abilities of both the non-corrected and corrected cell populations.

In this report, we establish a protocol that utilizes a thymidine treatment following the introduction of the oligonucleotide, allowing for a population wide reduction in DNA replication activity, regardless of correction state of the individual cells. The post-treatment led to a decrease in cell replication by an average of nearly 8 fold at each time point and as a result, correction levels remained constant. This observation was also true for cells not pre-treated with 1 mM thymidine prior to oligonucleotide electroporation (data not shown). In addition, removing the thymidine following ssODN electroporation at 48 hours and 72 hours allowed for the maintenance of substantial levels of correction up to 144 hours. The wash-out of the thymidine treatment was accompanied by the resumption of cellular replication and a normal division rate. This implies that thymidine can be used to slow the replication of the entire cell culture temporarily in order to preserve a high level of gene correction.

In contrast, treating the cells with 2 mM thymidine for 144 hours resulted in the population entering into cellular senescence, as determined by a physiological change in cell morphology and a positive staining for SA β-galactosidase. Interestingly, the development of senescence was time-dependent as removal of thymidine at 48 hours, but not 72 hours, in an assay lasting 144 hours showed a minimal number of senescent cells, indicating that there is a window of time in which the transition between survival and senescence is determined.

Normal cells can revert to a state of replicative senescence within several days, when exposed to certain physiological stress factors [see [47,48] and references therein]. In addition, hydroxyurea (HU), which depletes cells of dNTPs and rapidly shuts down cell replication [49,50], has been shown to induce senescence following a long treatment [51,52]. Not only could the entry into senescence after a prolonged treatment with thymidine be due to the cellular stress following an imbalance of ribonucleotide reductase, but also due to activation of other pathways that lead to cell senescence. Excess thymidine in the cell has been shown to activate an ATM-mediated protein kinase cascade which is followed by an ATR-mediated response; both of which are required for cell survival following the treatment [53]. The response is believed to emanate from an alteration in the chromatin state [54] or the formation of an altered DNA structure, known as a "chicken foot," following genome-wide stalling of replication forks [53,55-57], which p53 is able to recognize and bind to [58]. Both structures can activate ATM and along with a prolonged p53 induction, has been shown to mediate the onset of a senescent phenotype [59-61].

## Conclusion

Thus, based on these data, it is clear that the maintenance of gene conversion levels resulting in a 144 hour thymidine treatment may be due to the overall stalling in cell replication and an eventual entry into the senescent state. On the contrary, the wash out protocol at 48 hours was able to prevent this senescence transition followed by a reversal of the cell replication and division effects, and thus, may be a useful tool to increase and sustain high levels of gene repair.

## Methods

### Cell line and culture conditions

DLD-1 cells were purchased from American Type Cell Culture (ATCC, Manassas, VA). The integrated DLD-1 clone one (DLD-1-1) was obtained by integrating the pEGFP-N3 vector containing a single point mutation (TAG) in the chromophore region of the eGFP gene, and "clone one," containing 2–3 copies of the mutated eGFP, was isolated through serial dilution [see [18]]. For these experiments, cells were grown in RPMI 1640 medium supplemented with 2 mM glutamine, 4.5 g/l glucose, 10 mM HEPES, 1 mM sodium pyruvate, and supplemented with 10% FBS. Cells were maintained at 37°C and 5% CO_2 _and kept under selection by the addition of 200 μg/ml G418 to the media (Gibco, Invitrogen Co., Carlsband, CA).

### Oligonucleotide Designs, eGFP targeting, and flow cytometry analysis

EGFP3S/47NT (IDT, Coralville, IA) is single stranded DNA oligonucleotides 47 nucleotides long containing three phosphorothioate linkages at both the 5' and 3' ends. This oligonucleotide was designed to be complimentary to the non-transcribed strand and contains a mismatch at the site of the desired base change, which converts the mutant stop codon to the wild type TAC (encoding tyrosine), restoring the expression of eGFP in corrected cells. Previous publications have shown that neither a non-specific nor a perfectly matched oligonucleotide is able to foster this conversion [18].

For the eGFP targeting experiments, DLD-1 cells were subcultured at a density of 1.6 × 10^6 ^cells per 100 mm dish 24 hours prior to targeting. Following this incubation, cells were washed with PBS, trypsinized, centrifuged, and re-suspended in serum-free medium at a concentration of 2 × 10^6 ^cells/100 μl; 100 μl was transferred to a 4 mm gap cuvette (Fisher Scientific, Pittsburgh, PA). The EGFP3S/47NT oligonucleotide was added at a concentration of 1 μM and the cells were electroporated (LV, 250 V, 13 ms, 2 pulses, 1 s interval) using a BTX Electro Square Porator™ ECM830 apparatus (BTX, Holliston, MA). The cells were then transferred to a 60 mm dish (100 mm for incubations exceeding 48 hours) containing fresh medium supplemented with 10% FBS and incubated for the indicated time points at 37°C before harvesting for FACS or other analysis, as indicated.

eGFP fluorescence of corrected cells was measured by a Becton Dickinson FACS calibur flow cytometer (Becton Dickinson, Rutherford, NJ.). For eGFP fluorescence, cells were harvested after electroporation (between 8 and 144 hours, as indicated) and re-suspended in FACS buffer (.5% BSA, 2 mM EDTA, 2 μg/ml propidium iodide in PBS). 50,000 cells were analyzed for each sample and the cells that were eGFP positive (corrected) and PI negative (live) were scored as "corrected." Settings for the FACS calibur were determined as previously described [21].

### Thymidine treatment

Thymidine was obtained from Sigma-Aldrich (St. Louis, MO.) with a stock solution prepared in distilled water to a final concentration of 500 mM and added to incubating cells at a final concentration of 1–12 mM, as indicated. The term "pre-treatment" indicates addition of thymidine at the start of the 24 hour incubation prior to electroporation; "post-treatment" refers to the addition of thymidine to the 60 mm or 100 mm dishes following electroporation, in the recovery phase. Washing out of thymidine refers to the removal of the agent from the cell culture by aspirating off medium, washing the cells twice with PBS, and adding fresh medium containing FBS. For incubations exceeding 72 hours in the presence of thymidine, cells were washed twice with PBS and fresh medium was added in addition to thymidine at the 72 hour mark, to maintain desired thymidine concentration.

### BrdU cell replication assay, PKH26 cell division, and cell cycle analysis

BrdU, 5-Bromo-deoxyuridine, was obtained from Roche Applied Science (Indianapolis, IN). Cells were treated as for gene targeting and the time points indicated, 10 μM BrdU was added to the growth medium. Cells were incubated for 30 minutes at 37°C and then washed three times with PBS and DNA was denatured in 0.2 M HCl for 1 h at 37°C. After two washes in 0.1 M sodium borate and three washes in PBS, cells were blocked in PBS-0.5%BSA-0.1% Tween-20 for 20 min. Cells were then incubated for 1 hour at room temperature with Anti-BrdU-fluorescein antibody (Roche Diagnostics, Switzerland) diluted in PBS-0.1% BSA at a final concentration of 25 μg/ml.

PKH26 (MINI-26 Red Fluorescent Cell Linker Mini Kit) was obtained from Sigma-Aldrich (St. Louis, MO.) and the supplied protocol was optimized for the DLD-1 cells. About 5 × 10^6 ^cells were collected and washed once with PBS, cells were resuspended in 200 μl Diluent C, equivolume of PKH26 at 8 × 10^-6 ^M in Diluent C was added, and incubated at room temperature for 5 minutes, with occasional agitation. 400 μl 1% BSA was added for 1 minute at room temperature to stop the reaction. 800 μl DLD-1 medium containing FBS was added, cells were spun down at 1500 rpm for 5 minutes, medium was aspirated off and cells were re-suspended in 800 μl PBS and transferred to a clean tube. Cells were washed twice more with PBS and re-suspended in DLD-1 medium prior to plating. An aliquot was removed to determine mean fluorescence intensity (m.f.i.) immediately following staining (T0), the remaining cells were plated in the conditions noted and their fluorescence was measured at 48 or 144 hours (T48 and T144, respectively), as indicated. Detection of PKH26 was done via flow cytometry, using a rhodamine filter.

For cell cycle analysis, cells were treated as indicated, harvested and washed in PBS. They were then spun at 1200 rpm for 5 minutes, re-suspended in 500 μl cold PBS and fixed by adding 5 ml of 70% cold ethanol while vortexing. Following a 4°C incubation, the cells were centrifuged for 5 minutes at 2000 rpm, washed once with PBS and then re-suspended in 300 μl FACS buffer (50 μg/ml RNase A, 2.5 μg/ml PI, 1% FBS in PBS). The cells were incubated at 37°C for 1 hour and at 4°C over night before analysis by flow cytometry. DNA content was analyzed and data were plotted as DNA content versus cell number; cell cycle modeling was performed and the percent of cells in G_0_–G_1_, S, and G_2_-M phase was calculated using Modfit (Verity Software House, Topsham, ME.).

### Senescence associated β-Gal assay

At the indicated times, cells were washed twice with PBS and fixed with 4% paraformaldehyde for 10 minutes at room temperature. β-gal staining solution (40 mM citric acid/sodium phosphate, pH 6.0; 150 mM NaCl; 2 mM MgCl_2_; 5 mM potassium ferrocyonide; 5 mM potassium ferricyonide; 1 mg/ml X-gal) was added and plates were incubated over night at 37°C. Following this, cells were washed twice with PBS and re-suspended in PBS for microscopy studies. Cells were viewed under phase contrast and to enhance the blue staining, a bright field illumination was used. Images were obtained by a Nikon Coolpix 995 digital camera at 20× magnification.

## Abbreviations

Single-stranded oligonucleotides (ssODN)

## Authors' contributions

JE carried out all the experiments and in conjunction with EK, conceived the study and drafted the manuscript. All the authors have read and approved the final manuscript.

## Supplementary Material

Additional File 1 ***Gene repair at 8 hours following ssODN electroporation***. Cells were treated either with or without 1 mM thymidine for 24 hours prior to the electroporation of 1 μM EGFP3S/47NT and a subset of the samples was allowed to recover in the presence of 2 mM thymidine. Gene repair was assayed by flow cytometry at 8 hours following the introduction of the ssODN and correction is given as the percent of GFP(+)/PI(-) [fluorescing, live] cells out of the 50,000 cells analyzed. The data shows that the pre-treatment with 1 mM thymidine resulted in a greater than a 5 fold increase in gene repair (0.12% vs. 0.66%), as compared to the non-treated sample. The addition of 2 mM thymidine in the recovery led to a 3 fold decrease in repair frequency of the pre-treated sample (0.21% vs. 0.66%) but did not alter the correction levels of the no pre-treated sample (0.11% vs. 0.12%). Click here for file

Additional File 2 ***Cell cycle profile at 8 hours in the presence of a 2 mM thymidine post-treatment***. Cells were cultured for 24 hours in the presence or absence of a 1 mM thymidine pre-treatment, electroporated with 1 μM of the oligonucleotide and then recovered in 2 mM thymidine. After an 8 hour recovery period, the cells were trypsinized and fixed to be processed for cell cycle analysis, as outlined in the methods section. When cells were not pre-treated with thymidine, the post-treatment did not alter the cell cycle profile (G1: 59%; S: 27%; G2/M: 14%). However, when the post-treatment followed the 1 mM thymidine pre-treatment, a considerable percent of cells remained in G1 or early S (G1: 62%; S: 36%; G2: 2%). Click here for file
