# Supplementary figures and images for: Manipulation of cell cycle progression can counteract the apparent loss of correction frequency following oligonucleotide-directed gene repair
Source: BMC Mol Biol. 2007 Feb 6;8:9. doi: 10.1186/1471-2199-8-9 (PMC1797188; doi:10.1186/1471-2199-8-9)

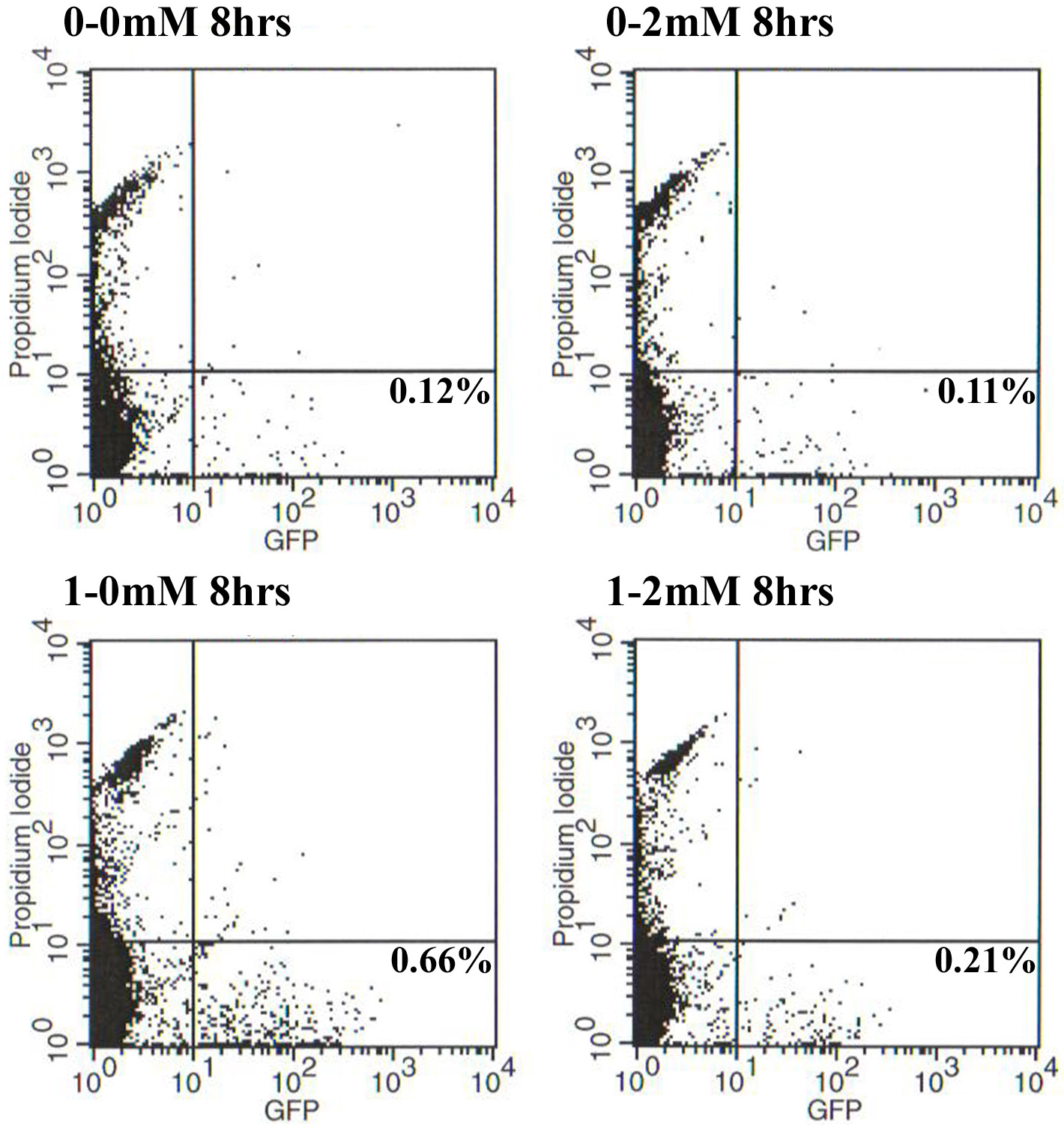

Supplement: Additional File 1 — Gene repair at 8 hours following ssODN electroporation. Cells were treated either with or without 1 mM thymidine for 24 hours prior to the electroporation of 1 μM EGFP3S/47NT and a subset of the samples was allowed to recover in the presence of 2 mM thymidine. Gene repair was assayed by flow cytometry at 8 hours following the introduction of the ssODN and correction is given as the percent of GFP(+)/PI(-) [fluorescing, live] cells out of the 50,000 cells analyzed. The data shows that the pre-treatment with 1 mM thymidine resulted in a greater than a 5 fold increase in gene repair (0.12% vs. 0.66%), as compared to the non-treated sample. The addition of 2 mM thymidine in the recovery led to a 3 fold decrease in repair frequency of the pre-treated sample (0.21% vs. 0.66%) but did not alter the correction levels of the no pre-treated sample (0.11% vs. 0.12%). [file 1471-2199-8-9-S1.jpeg]

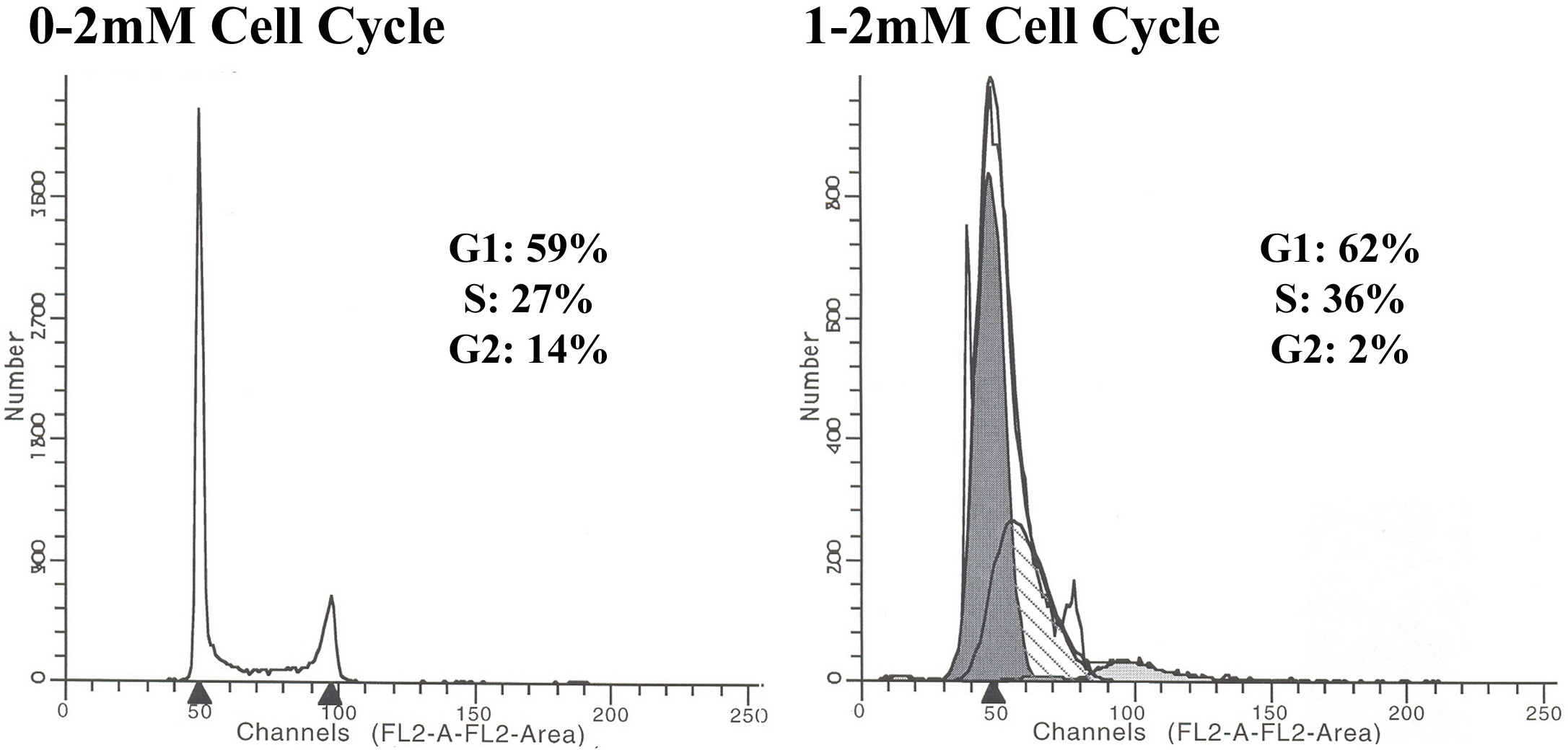

Supplement: Additional File 2 — Cell cycle profile at 8 hours in the presence of a 2 mM thymidine post-treatment. Cells were cultured for 24 hours in the presence or absence of a 1 mM thymidine pre-treatment, electroporated with 1 μM of the oligonucleotide and then recovered in 2 mM thymidine. After an 8 hour recovery period, the cells were trypsinized and fixed to be processed for cell cycle analysis, as outlined in the methods section. When cells were not pre-treated with thymidine, the post-treatment did not alter the cell cycle profile (G1: 59%; S: 27%; G2/M: 14%). However, when the post-treatment followed the 1 mM thymidine pre-treatment, a considerable percent of cells remained in G1 or early S (G1: 62%; S: 36%; G2: 2%). [file 1471-2199-8-9-S2.jpeg]
